# FastCloning: a highly simplified, purification-free, sequence- and ligation-independent PCR cloning method

**DOI:** 10.1186/1472-6750-11-92

**Published:** 2011-10-12

**Authors:** Chaokun Li, Aiyun Wen, Benchang Shen, Jia Lu, Yao Huang, Yongchang Chang

**Affiliations:** 1 Division of Neurobiology, Barrow Neurological Institute, St. Joseph's Hospital and Medical Center, Phoenix, AZ, USA; 2 Department of Obstetrics and Gynecology; St. Joseph's Hospital and Medical Center, Phoenix, AZ, USA; 3 Department of Genetics and Cell Biology, Guangzhou Medical University, Guangzhou, China

## Abstract

**Background:**

Although a variety of methods and expensive kits are available, molecular cloning can be a time-consuming and frustrating process.

**Results:**

Here we report a highly simplified, reliable, and efficient PCR-based cloning technique to insert any DNA fragment into a plasmid vector or into a gene (cDNA) in a vector at any desired position. With this method, the vector and insert are PCR amplified separately, with only 18 cycles, using a high fidelity DNA polymerase. The amplified insert has the ends with ~16-base overlapping with the ends of the amplified vector. After *Dpn*I digestion of the mixture of the amplified vector and insert to eliminate the DNA templates used in PCR reactions, the mixture is directly transformed into competent *E. coli *cells to obtain the desired clones. This technique has many advantages over other cloning methods. First, it does not need gel purification of the PCR product or linearized vector. Second, there is no need of any cloning kit or specialized enzyme for cloning. Furthermore, with reduced number of PCR cycles, it also decreases the chance of random mutations. In addition, this method is highly effective and reproducible. Finally, since this cloning method is also sequence independent, we demonstrated that it can be used for chimera construction, insertion, and multiple mutations spanning a stretch of DNA up to 120 bp.

**Conclusion:**

Our FastCloning technique provides a very simple, effective, reliable, and versatile tool for molecular cloning, chimera construction, insertion of any DNA sequences of interest and also for multiple mutations in a short stretch of a cDNA.

## Background

Molecular cloning is one of the most widely used techniques in biomedical research laboratories. Traditionally, molecular cloning joins insert and vector by T4 DNA ligase after restriction digestion to excise insert from a donor vector or from a PCR product with restriction enzyme recognition sites added to the ends [1]. Although this is a widely used method, it involves multiple steps and is time consuming. This multi-step process also makes it difficult or complicated for troubleshooting. To overcome the difficulties encountered in the original cloning method, many other alternative cloning methods have been developed over the last two decades. These methods include TA cloning [2], ligation independent cloning with T4 DNA polymerase [3,4], GATEWAY recombinational cloning [5], and more recent sequence- and ligation-independent cloning kits, such as CloneEZ (GenScript USA Inc., Piscataway, NJ, USA), one step cloning [6], and overlap extension PCR cloning [7]. However, each of these techniques has its own limitations. For example, TA cloning uses regular Taq DNA polymerase to add a single 3'-A overhang to the ends of the PCR product. The PCR product is directly cloned into a TA cloning vector with a complementary 3'-T overhang in both ends without restriction digestion. The limitations of this method are low fidelity of Taq DNA polymerase causing unwanted mutations and requirement of subcloning into the final target vector with restriction digestion and ligation. The early ligation independent cloning uses the 3'-exonucnease activity of T4 DNA polymerase to create 15-base 5'overhangs in the ends of insert and complementary 5' overhangs in the ends of vector. This technique requires specific sequences to create 15-base overhangs. Gateway recombinational cloning uses site-specific recombination to transfer cDNAs between donor and destination vectors, which requires additional specific sequences for recombination. The latest ligation-independent cloning, such as CloneEZ and In-Fusion cloning kits, uses some DNA polymerase to generate sticky ends in the vector and insert without specific sequence requirement, except for restriction sites to linearize the vector. However, the new ligation independent cloning still requires purification of the digested vector and PCR-amplified insert, and the purchase of purification and cloning kits. Similarly, overlap extension PCR cloning also requires purification of the first round PCR products (vector and insert) and an additional round overlap extension PCR, which usually generates multiple bands, for producing linked vector and insert. One-step "quick assemble" cloning does not need purification of PCR products. However, it includes two sequential 35-cycle PCRs with a total number of 70 cycles. The first-round PCRs are used to amplify insert and linear vector. The second-round PCR is essentially the overlap extension PCR to assemble vector and insert into a single linear PCR product. Another ligation independent cloning technique, using nick DNA endonuclease to create long single-strand 5' overhangs in the vector and PCR-amplified insert [8], requires specific sequences for nick DNA endonuclease and purification of the PCR product.

Purification of PCR product not only takes extra time and requires purification kit, but also potentially creates additional problems. For example, to compensate for the loss of PCR product during purification, the number of PCR cycles is generally 25-30. High number of PCR cycles increases the chance of random mutations or, in our experiences, dramatically decreases cloning efficiency for the PCR products generated by high fidelity DNA polymerases. This is especially true for the enzyme with high processability, such as pfuUltraII DNA polymerase. The decrease in cloning efficiency cannot be completely overcome by using state-of-the-art cloning kits, such as CloneEZ. Using this kit (with gel purification of digested vectors and PCR-amplified inserts), the successful rate in our laboratory still varies significantly with overall successful rate less than 50%. In the PCR-based QuickChange mutagenesis, the number of PCR cycles is recommended to be 12-18. Increase in PCR cycles could decrease efficiency according to the QuickChange protocol. Thus, these phenomena made us believe that the proofreading PCR enzymes could potentially damage the ends of PCR products if the number of PCR cycles increases. This could be due to the fact that the high fidelity DNA polymerases with the proofreading ability have 3' exonuclease activity. In the presence of dNTPs, the ends of PCR products can be protected from this 3' exonuclease activity. It is possible that depletion of dNTPs in high number of PCR cycles or dilution of dNTPs in the early stage of purification would weaken the 3' ends protection against 3' exonuclease activity of the DNA polymerase, which results in the damage of PCR products and makes cloning extremely difficult.

To circumvent the above-mentioned problems, we developed a cloning method termed FastCloning. With this method, both insert and vector are amplified by 18 PCR cycles with a high fidelity DNA polymerase. The unpurified PCR products of the vector and insert are then directly mixed at some ratios (see below) and digested by *Dpn*I restriction enzyme to destroy their methylated DNA templates. Finally, the digested mixture is transformed into competent cells to obtain the target clones. The PCR amplification of vector is designed to make it possible to clone an insert into any position of the vector, to bypass vector digestion (and restriction site limitation) and purification steps, and to be compatible with *Dpn*I digestion of the insert without further inactivation of the enzyme. Thus, this method can be used to construct cDNAs of fusion proteins or chimera without limitation by available restriction sites. In this study, we experimentally validated all these applications with our new method. In addition, a similar method can be easily adapted for deletion of a DNA fragment.

## Methods

**Figure 1:**
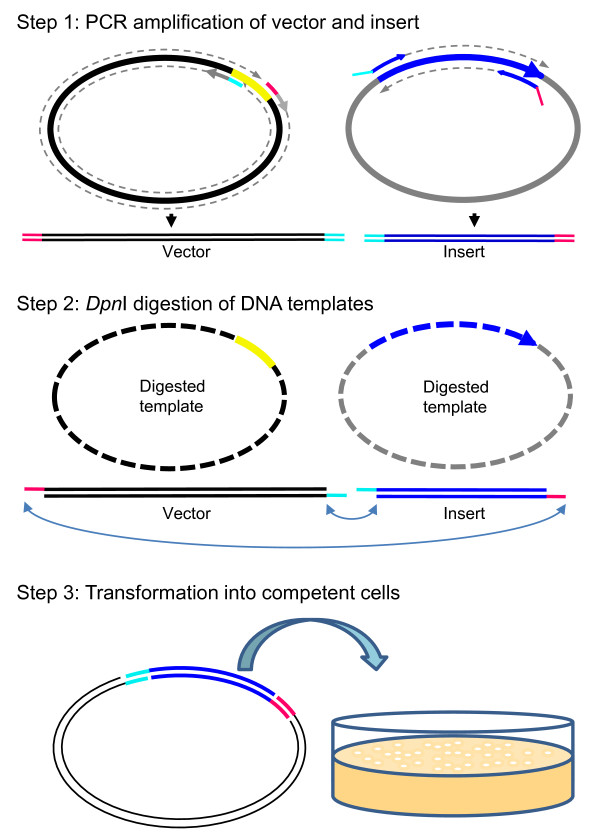
**The procedures for FastCloning: **Step 1. PCR amplification of vector and insert. Note that the primer pair for insert amplification has 16-base tails overlapping with the PCR-amplified vector ends. Step 2. *Dpn*I digestion. The parent DNA templates (if in a plasmid) for PCR amplification needs to be methylated in order to be compatible to *Dpn*I digestion. Although the detailed mechanism is not known, it is likely that the 3' exonuclease activity of the high fidelity DNA polymerase directly creates sticky ends for the overlapped regions of the vector and insert during *Dpn*I digestion, allowing them to form a circular construct with nicks. Step 3. transformation into competent *E. coli*. cells. The nicks will be repaired after transformation into the bacteria.

Figure 1 illustrates the highly simplified procedure of our FastCloning method. Briefly, after gel confirmation of PCR products, the remaining unpurified PCR reactions, containing amplified vector and insert, are mixed and digested with *Dpn*I for 1 hour at 37°C. The digested mixture is then directly transformed into chemically competent Stratagene XL-10 Gold or NEB 10-beta *E. coli *cells.

The primers were designed with Oligo Analyzer 1.5 (http://www.genelink.com) to have an annealing temperature around 60°C (Nearest Neighbor method). The forward primer for vector amplification is in the 3' side of the polylinker region. The reverse primer for vector amplification is in the 5' side of the polylinker, and its reverse and complementary sequence was generated by Oligo Explorer 1.5. The primers for insert amplification have insert-specific sequences and additional 15-17 bases (depending on the GC content) overlapping with the vector ends. The specific sequences of primers used in this study are listed in Table 1. All the primers used in this study were synthesized by Invitrogen Corporation (Carlsbad, CA).

**Table 1:** Primers used in the cloning or mutagenesis experiments

Primer name	Primer description	Primer sequence
pGEMHEStartRev	Reverse primer for pGEMHE vector with start codon	CATGGCCAAAGTTGAGCGTTTATTCTG
pGEMHEStopFwd	Forward primer for pGEMHE vector with stop codon	TAAACCAGCCTCAAGAACACC
p3X14startRV	Reverse primer for p3xFlag-cmv-14 vector with start codon	CATGGTGGCGAATTCGCGGCCGCAAGC
p3X14endFW	Forward primer for p3xFlag-cmv-14 vector	GGATCCCGGGCTGACTAC
CHRNA9Startez	Forward primer for *CHRNA9 *cloning into pGEMHE	CTCAACTTTGGCCATGAACTGGTCCCATTCCTGCAT
CHRNA9Stopez	Reverse primer for *CHRNA9 *cloning into pGEMHE	TCTTGAGGCTGGTTTAATCCGCTCTTGCTATGATCAAA
CHRNB2qk5	Forward primer for *CHRNB2 *cloning into pGEMHE	GCTCAACTTTGGCCATGGCCCGGCGCTGCGG
CHRNB2qk3	Reverse primer for *CHRNB2 *cloning into pGEMHE	TTCTTGAGGCTGGTTTACTTGGAGCTGGGGGCTG
CHRNA4_qk5	Forward primer for *CHRNA4 *cloning into pGEMHE	GCTCAACTTTGGCCATGGAGCTAGGGGGCCC
CHRNA4_qk3	Reverse primer for *CHRNA4 *cloning into pGEMHE	TTCTTGAGGCTGGTTTAGATCATGCCAGCCAGCC
ECSM2_qk5	Forward primer for *ECSCR *cloning into pGEMHE	GCTCAACTTTGGCCATGGGCACCGCAGGAGC
ECSM2_qk3	Reverse primer for *ECSCR *cloning into pGEMHE	TTCTTGAGGCTGGTTTAAAGAACCTTCTCTGCTGAGAG
HEstartFW-p3X14	Forward primer for *ECSCR *cloning into p3xFlag-cmv-14	CGAATTCGCCACCATGGGCACCGCAGGAGC
HEendRV-p3X14	Reverse primer for *ECSCR *cloning into p3xFlag-cmv-14	AGTCAGCCCGGGATCCAAGAACCTTCTCTGCTGAGAG
ZACNqk5	Forward primer for *ZACN *cloning into pGEMHE	GCTCAACTTTGGCCATGATGGCCCTATGGTCCCTG
ZACNqk3	Reverse primer for *ZACN *cloning into pGEMHE	TTCTTGAGGCTGGTTTACAGTCTAGGCCGCCTGC
APBB1qk5	Forward primer for *APBB1 *cloning into pGEMHE	GCTCAACTTTGGCCATGTCTGTTCCATCATCACTGAG
APBB1qk3	Reverse primer for *APBB1 *cloning into pGEMHE	TTCTTGAGGCTGGTTTATGGGGTATGGGCCCCCA
HTR3A5P381_5A5	Forward primer of *HTR3A *for 5 Pro mutated to Ala	CGCTGCCGCAGCAGCTCGGGAGGCCTCGCTG
HTR3A5P381_5A3	Reverse primer of *HTR3A *for 5 Pro mutated to Ala	GAGCTGCTGCGGCAGCGCTACATCTGTCCCTCGGG
pLXSN3'fwd	Forward primer for pLXSN vector	GGATCCGGCTGTGGAATGTG
pLXSN5'rev	Forward primer for pLXSN vector	GAATTCCGGCGCCTAGAGAA
Akt3v1&2Start	Forward primer for Akt3v1 & v2 with Kozak sequence	CTAGGCGCCGGAATTCCATCATGAGCGATGTTACCATTGTG
Akt3v1StopRev	Reverse primer for Akt3v1 cloning into pLXSN	TTCCACAGCCGGATCCTTATTCTCGTCCACTTGCAGAGTAG
Akt3v2StopRev	Reverse primer for Akt3v2 cloning into pLXSN	TTCCACAGCCGGATCCTTATTTTTTCCAGTTACCCAGCATGC
β2β4N_Rv	Reverse primer for vector along with N-terminal cDNA	GATGATGAGGTTGATGGTGTAG
β2β4C_Fw	Forward primer for insert amplification	CATCAACCTCATCATCCCCTG
β2StopRv_HE	Reverse primer for C-terminal β2 insert amplification	CTTGAGGCTGGTTTACTTGGAGCTGGGGGCTG
β4StopRv_HE	Reverse primer for C-terminal β4 insert amplification	CTTGAGGCTGGTTTAGTCACGCTGGGCAGCGT
AChBPins1Fw	Forward primer for AChBP insert1 to α7 nAChR	CCCGGCCCCACCAAGGACGACCCCCTGACCGTGACCCTGGG CTTCACCCTGCAGGACATCATGGACGT**G**GATGAGAAGAAC
AChBPins1Rv	Reverse primer for AChBP insert1 to α7 nAChR	CTTGGTGGGGCCGGGGTACATGGGGCTTCTGTTGAACA GGTCGCTCTTCAGTCTCATCTGGAACTCGCCTTGCAGG
AChBPins2Fw	Forward primer for AChBP insert2 to α7 nAChR	TGTGGGACCCCAACGAGTACGGCAACATCACCGACTTCAGA ACCAGCGCCGCCGACATTTGGAAACCAGACATTCTTCTCTA
AChBPins2Rv	Reverse primer for AChBP insert2 to α7 nAChR	TCGTTGGGGTCCCACATCAGGCTGTTCAGCTTCCATCTCT GCTGCTCGTAGTACACGGTGGTTAAAACTTGGTTCTTCTC
AChBPins3Fw	Forward primer for AChBP insert3 to α7 nAChR	TCGCCGTGGTGACCCACGACGGCAGCGTGATGTTCATCCCCG CCCAGAGACTGAGCTTCATGTGCTACATCGATGTACGCTGG
AChBPins3Rv	Reverse primer for AChBP insert3 to α7 nAChR	TGGGTCACCACGGCGATCTGGGGGCTCAGCACCTGCACGGGTC TGGTGCTGCTGTAGGCGGTAATGTCTGGTTTCCAAATGTCGG
AChBPins4Fw	Forward primer for AChBP insert4 to α7 nAChR	CTTCGAGATCGACCTGAAGACCGACACCGA CCAGGTGGATATCAGTGGCTATATCCCCA
AChBPins4Rv	Reverse primer for AChBP insert4 to α7 nAChR	CAGGTCGATCTCGAAGCCGCTGTACACCCAGCTG CCGAACTTCACGGCGCAGTGCTGCACATCAAAGG
AChBPins5Fw	Forward primer for AChBP insert5 to α7 nAChR	TACAGCTGCTGCCCCGAGCCCTACATCGACGTGAA CCTGGTGGTGAAGTTCCGCCGCAGGACACTCTAC
AChBPins5Rv	Reverse primer for AChBP insert5 to α7 nAChR	CGGGGCAGCAGCTGTAGTGCTGCACCTGTCTGGTCT GGGTGGCGCTCAGGATGTCCCATTCTCCATTGGGGA

The PCR reaction components were: 50 μl total volume, 0.5 μl Phusion DNA polymerase (New England Biolabs, Ipswich, MA), or 0.8 μl *Pfu *Turbo, or PfuUltra DNA polymerase (Agilent Technologies, Inc, Santa Clara, CA), 5 μl 10× buffer; 5 μl of 2.5 mM dNTPs; 10 ng of plasmid DNA template; 5 pmol of each primer. The PCR cycling parameters were 98°C 3 min, (98°C 10 sec, 55°C 30 sec, 72°C 20 sec/kb) × 18 cycles, 72°C 5 min, and 4°C infinite for Phusion DNA polymerase, and 95°C 3 min, (95°C 15 sec, 55°C 1 min, 72°C 1 min/kb) × 18 cycles, 72°C 5 min, and 4°C infinite for *Pfu *Turbo or PfuUltra DNA Polymerase. The PCR products (5 μl for each product) were examined with 1% agarose gel electrophoresis with ethidium bromide staining using VWR Mini Gel electrophoresis setup (VWR International, Marietta, GA, USA) running at 100 V for 30 min. The PCR products were then visualized under a UV transilluminator, and gel pictures were taken using an AGFA scanner.

After confirmation of PCR products, 1 μl of *Dpn*I enzyme (New England Biolabs) was added into the remaining unpurified PCR reactions (45 μl for each product) for vector or insert separately. The vector and insert were then mixed with 1:1 ratio (1:1, 1:2, and 1:4 for α9 nAChR subunit), and digested at 37°C for 1 hour. Two micro-liters (2, 4, and 8 μl for α9 nAChR) of the digested vector-insert mixture were then added to 40 μl of chemically competent XL-10 Gold *E. coli *cells (prepared with rubidium chloride method) unless indicated otherwise. The mixture was then incubated for 30 min on ice. After heat shock at 42°C for 45 sec, 350 μl of SOC medium was added to the mixture. After 60 min shaking at 37°C and 350 rpm with an Eppendorf Thermomixer, the entire content was plated onto the LB agar plate containing 100 μg/ml ampicillin. The plates were then incubated at 37°C overnight. Next day, colonies from each constructs were picked for PCR confirmation of each construct using GoTaq DNA polymerase (Promega, Madison, WI, USA) and vector specific primers, and also for inoculation in the LB medium (with ampicillin) for overnight culture of each clone for mini-prep. The DNA mini-prep was performed using QIAprep Spin Miniprep Kit (QIAGEN, Valencia, CA, USA). All the cloned sequences were finally confirmed by automated DNA sequencing at the DNA lab of the Arizona State University using primers in the vectors.

**Figure 2:**
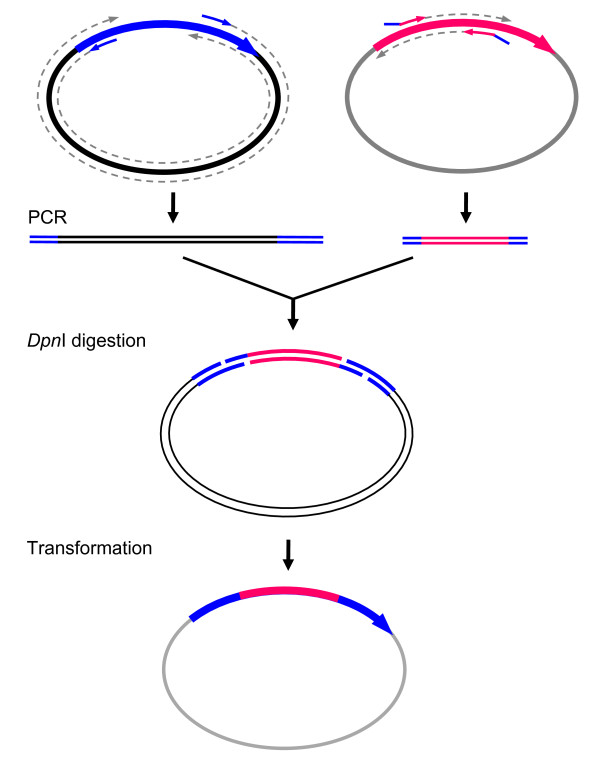
**Chimera construction or insertion**. *Top left*: PCR amplification of both ends of the parent cDNA along with the vector. For chimera construction, one fragment of the cDNA needs to be replaced. Thus, the forward primer is immediately downstream of the fragment to be substituted, and the reverse primer will be immediately upstream of the fragment to be substituted. For insertion, however, two primers will be next to each other without skipping a single base. *Top right: *Insert amplification of the equivalent region of a homologous gene (cDNA) for chimera construction. However, for insertion of a cDNA encoding a full length protein, such as green fluorescent protein, the insert amplification will cover the entire cDNA. The remaining procedure is the same as in Figure 1.

Figure 2 illustrates the application of this method to construct cDNAs encoding chimeric or fusion proteins. In this case, the PCR amplification of the vector also includes part of the cDNA in both ends. To insert a cDNA encoding a full-length protein, such as green fluorescence protein, the insert amplification should cover the entire coding region of the cDNA. For homologous domain swap chimera, insert amplification only needs to cover the corresponding region of a cDNA encoding a homologous fragment of protein. The detailed experimental procedures for chimera construction are essentially the same as cloning.

**Figure 3:**
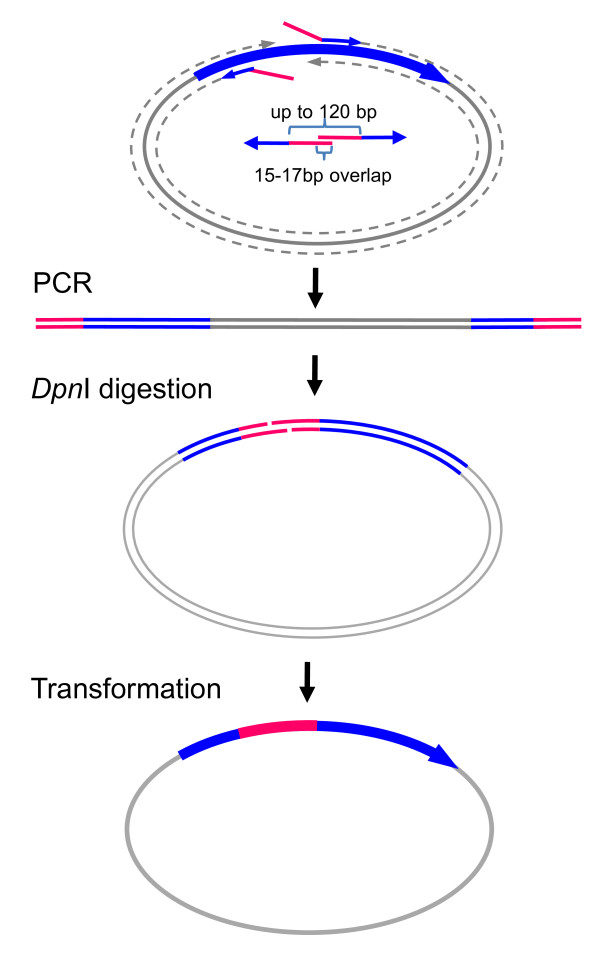
**Chimera construction or insertion with a short DNA fragment**. To replace a short stretch of DNA, such as a DNA fragment encoding a transmembrane region of a nAChR subunit for chimera construction, or to insert a short tag, such as FLAG-tag, into some part of a protein, amplification of the insert is not necessary. In this case, the insert can be directly included in two primers for single PCR amplification of the cDNA along with the vector. The forward primer starts immediately downstream of the insertion site and has a tail with the 3' part of the insert. The reverse primer starts immediately upstream of the insertion site and has a tail with 5' part of the insert. Two primers only require ~16-base overlap. Thus, for a 120 bp insertion, each primer needs to have a 68-base tail for insertion and ~17-22 bases for annealing (depending on the GC content). The total length of each primer will be about 85-90 bases. *Dpn*I digestion and transformation are the same as in Figure 1.

For insertion (or substitution) of a short DNA fragment (< 120 bp), the insert can be directly included in the primer sequences for vector amplification (Figure 3). This is a very convenient way to insert a short tag (such as myc tag or FLAG-tag) or to replace a short DNA fragment for chimera construction without limitation of the availability of specific DNA template for the insert. Introducing multiple mutations in a short (up to 120 bp) stretch of a cDNA is equivalent to replacing a short DNA fragment for chimera construction.

## Results and Discussion

As a proof of principle, we subcloned several cDNAs into different vectors (human nicotinic receptor (nAChR) α4, α9 and β2 subunits, and serotonin receptor type 3A (5-HT_3_A) subunit into the pGEMHE vector, human endothelial cell-specific molecule 2 (ECSM2) into the p3XFLAG-CMV-14 vector, and Akt3v1 or Akt3v2 into pLXSN vector). pGEMHE is a vector containing 5' and 3' untranslated regions (5'UTR and 3'UTR) of *Xenopus *β-globin, which is highly expressed in *Xenopus *oocytes. With this vector, the protein expression level of the inserted gene in *Xenopus *oocyte can be increased by up to 200-fold [9].

**Figure 4:**
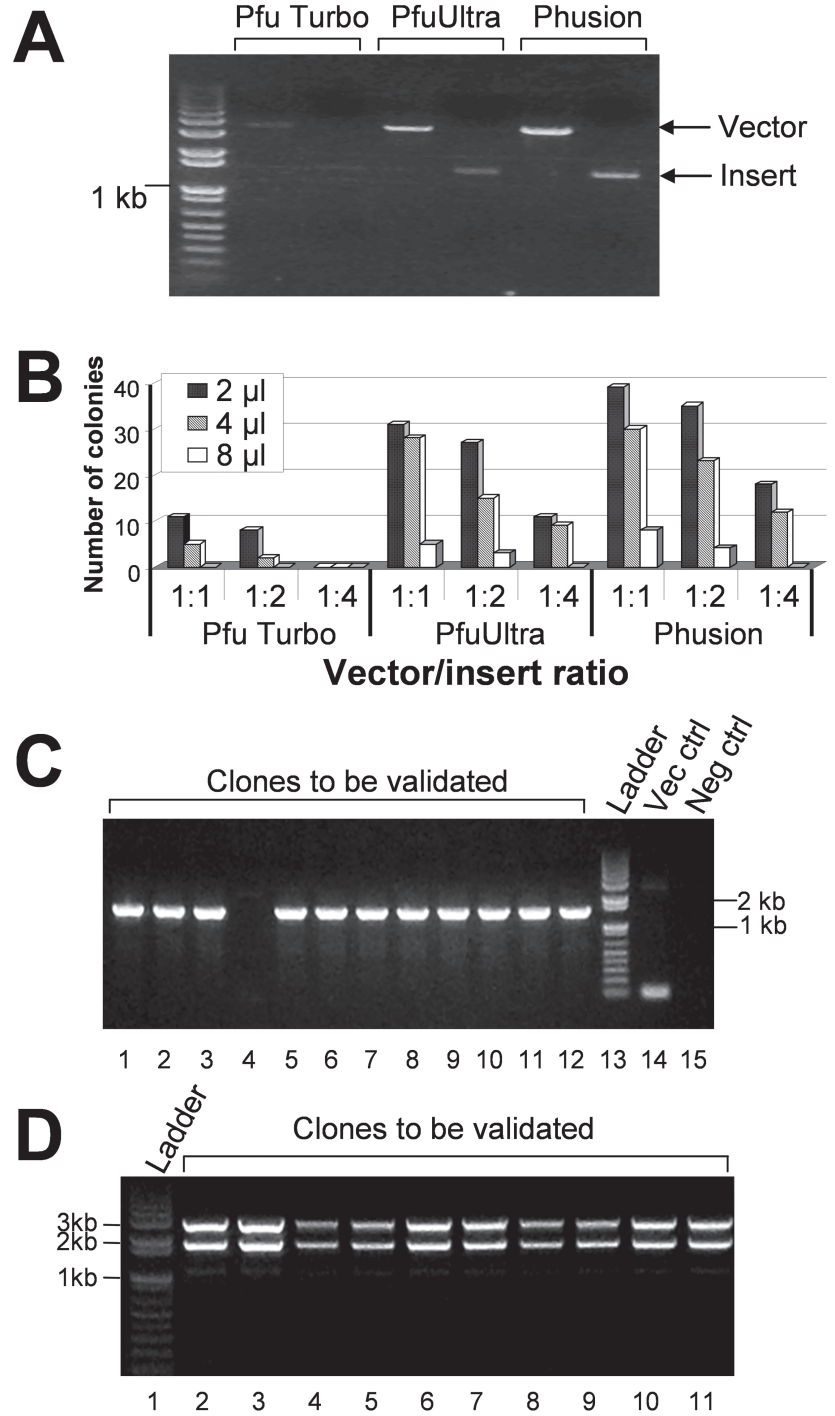
**Optimization of cloning conditions**. (A) PCR amplification of a target cDNA (human nAChR α9 subunit) and the pGEMHE vector using different DNA polymerases: *Pfu *Turbo, PfuUltra and Phusion. (B) Comparison of number of colonies grown on the plates after transformation. Three different vector-to-insert ratios (1:1, 1:2, and 1:4) during *Dpn*I digestion and three amounts of vector-insert mixtures (2, 4, and 8 μl) for transformation were tested. See text for details. (C) Clone validation by PCR using GoTaq DNA polymerase. Lanes 1 to 12: target clones to be validated; Lane 13: 1 Kb plus DNA ladder; Lane 14: pGEMHE vector control; Lane 15: negative control using pCR4-TOPO-α9 parent plasmid. (D) Clone validation by restriction digestion to exclude unusual constructs. Lane 1: 1 Kb plus DNA ladder, Lanes 2-11: target clones double digested with *Kpn*I and *Nhe*I. Note that this digestion resulted in a pGEM vector and an insert with α9 nAChR plus the 5'UTR and 3'UTR of *Xenopus *β-globin.

We first optimized cloning conditions by cloning α9 nAChR subunit cDNA into the pGEMHE vector. Figure 4A is an example of agarose gel electrophoresis for verification of PCR products of the vector (pGEMHE) and insert (α9 nAChR). In each lane, 5 μl of PCR products was loaded. Note that amplification efficiency is the highest for Phusion DNA Polymerases and lowest for *Pfu *Turbo. We then compared cloning efficiency, in terms of number of colonies produced after transformation, for optimal ratio of vector and insert during *Dpn*I enzyme digestion and optimal amount of the vector-insert mixture used in transformation. Vectors and inserts were mixed with three different ratios (1:1, 1:2, and 1:4 with a total volume of 16 μl), and incubated at 37°C for 1 h. Different amounts (2, 4, and 8 μl) of the *Dpn*I-digested mixtures were then added into 40 μl of chemically competent XL-10 Gold *E.coli *cells for transformation. The number of colonies that grew on each plate was counted next day. Figure 4B shows the number of colonies that resulted from different DNA polymerases, vector to insert ratios (1:1, 1:2, and 1:4), and the amount of vector-insert mixture (2 μl, 4 μl, and 8 μl) used for transformation. The results suggest that the best combination for colony formation is Phusion DNA polymerase amplification with 1:1 vector/insert ratio in *Dpn*I digestion and 2 μl of vector-insert mix for transformation. Four colonies from the plates resulting from each DNA polymerase were picked up for DNA mini-prep. The PCR confirmation of the insert in the target vector (Figure 4C) was performed with the GoTaq DNA polymerase (Promega Corporation, Madison, WI) using the pGEMHE vector specific primer pair. Our results indicated that > 90% colonies (11 out of 12) were positive. We have also compared colony production efficiency for three different numbers of PCR cycle (12, 18, and 24 cycles) and three different durations (1, 2, and 4 hours) of incubation time for 37°C *Dpn*I digestion with α9 nAChR cDNA cloned into pGEMHE and transformed into NEB 10-beta high efficiency competent *E coli*. The result shows that the 18-cycle PCR amplification of vector and insert produced many more colonies than the 12-cycle and 24-cycle amplifications (31, 302, 43 colonies for 12, 18, and 24 cycles respectively). For incubation duration in *Dpn*I digestion, 1, 2, and 4 hours of incubation had similar colony production efficiency with colony numbers of 281, 263, and 285 for 1, 2, and 4 hour-incubations, respectively). However, shorter incubation time is not recommended. In our QuickChange mutagenesis experiment, 30 min incubation in *Dpn*I digestion could not adequately remove the wild-type template background and resulted in a significantly higher fraction of wild type constructs.

In addition, the PCR-based method could produce some unusual constructs. In our > 10-year practice with the QuickChange site-directed mutagenesis, we only encountered an unusual recombination once. With our FastCloning method in the past few months, we have not seen any unusual construct yet. Thus, if our cloning method produces unusual constructs, their occurrence must be very low. Figure 4D is an example of using restriction digestion to screen clones to exclude unusual constructs. It would be a good practice to perform such a screening for all resulting clones before DNA sequencing. With the optimized cloning conditions (Phusion DNA polymerase, 1:1 vector/insert ratio, 18 cycle PCR amplification, 2 μl of vector-insert mix for transformation, and 1 hour *Dpn*I digestion), we have also successfully cloned other cDNAs into the target vectors, and obtained positive clones at efficiency ranging from 43% to 100%, with an overall efficiency > 70%. The results further validate the versatility and reliability of this new technique.

**Figure 5:**
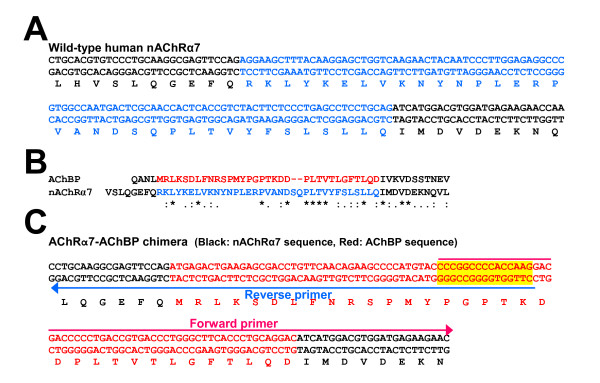
**An example of chimera construction with a short (105 bp) DNA fragment replaced by a synthesized insert of 99 bp (included in two primers)**. (A) A region of α7 nAChR subunit sequence with a 105 bp fragment (blue) to be replaced. (B) Amino acid sequence alignment of human α7 nAChR and corresponding region of *Aplysia californica *AChBP. Segments to be replaced are colored with blue (35 codons) for α7 nAChR sequence and red (33 codons) for AChBP sequence. (**C**) Target chimera construct with the human nAChR α7 subunit sequence (black) and a substituted 99 bp DNA fragment (red) from the mammalian codon-optimized homologous sequence of the AChBP. Two colored arrows indicate two primers with a 15 bp overlapping region (highlighted). Note that the entire insert of the 99 bp fragment is included in the two primers. Thus, there is no need to amplify the insert. The length of each primer is 81 bases for the forward primer and 76 bases for the reverse primer.

Because the PCR amplification of vector can be controlled by primers to exact positions, our FastCloning method is truly sequence-independent. Thus, one can put an insert to any position and in any frame. This feature, although with only a small modification of standard cloning protocol, makes it easy to construct cDNAs for fusion proteins or chimeras. Furthermore, a minor variation of this technique can be applied for insertion of a short DNA fragment directly from two relative long primers for PCR amplification of a cDNA along with its vector. As a proof of principle, we have successfully created human nAChR cDNAs encoding β2-β4 chimeric subunit proteins with C-terminal domain swap between the β2 and β4 subunits. We have also successfully inserted DNA fragments into cDNAs using two long primers with a 16-base overlapping region to directly amplify the cDNA. Figure 5 is an example of using a synthesized insert (99 bp), a DNA sequence encoding a fragment of an acetylcholine binding protein (AChBP), to replace a DNA fragment in the cDNA of the human α7 nAChR for chimera construction. Note that each primer contains only slightly more than half of the insert. The primer pairs have a 16-base overlap region. Using similar method, we have successfully replaced other four DNA fragments (105 bp, 90 bp, 69 pb, and 87 pb) in human α7 nAChR with synthetic AChBP fragments and swapped the DNA sequences (66 bp) encoding the second transmembrane domains of human α4 and β2 nAChR subunits.

**Figure 6:**
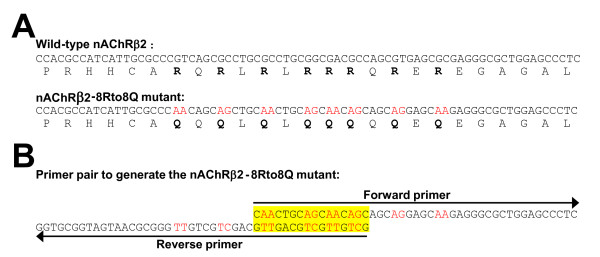
**An example of making multiple mutations across a wide region**. (A) A fragment of the human nAChR α2 subunit cDNA with 8 arginine (R, in bold) codons (top) to be substituted by glutamine (Q) codons (bottom). Mutated nucleotides are indicated in red. (B) Actual forward (45-base) and reverse (46-base) primers with 16 bp overlapping in their 5' ends.

Making multiple mutations in a stretch of DNA is equivalent to making short insertions. Figure 6 is an example of 8 amino acid substitutions (8 arginines to 8 glutamines) with multiple nucleotide mutations spanning a 13-codon region in the cDNA encoding human nAChR β2 subunit. With this relatively short primer pair (45-base forward primer and 46-base reverse primer), we have successfully obtained this multiple mutations spanning a 13-codon stretch in the cDNA. In addition, we have successfully made a quintuple mutant (5 consecutive prolines mutated to 5 alanines) of human 5-HT_3_A receptor subunit with a 31-base forward primer and 35-base reverse primer.

It is important to be aware that with PCR-based cloning, the synthesized primers may not be completely uniform with correct sequences. Random single-base deletions, mutations, or insertions can occur in a small fraction of primers, which result in unwanted deletion/mutation/insertion in a small fraction (< 5%) of clones. This is especially true for short insertion with long primers. Thus, sequencing across the primer region is required for ultimate confirmation of each clone. If random mutation happens in one clone, picking up another clone often solve the problem. The random mutation out of primer region is rare with high fidelity DNA polymerases. However, DNA sequencing of the entire coding region is still necessary. In the past 8 months, we have successfully used our new cloning method to obtain 21 constructs. Among the 63 sequenced clones, we found 2 deletions in 2 chimeras constructed with long primer pairs, but found no mutations in the entire regions of all constructs beyond primers. Finally, the cloning efficiency of our FastCloning method is high. Of the 21 cloning constructs, we obtained 19 desired constructs in single runs. In 2 experiments, we needed to repeat the procedure to get the final clones. With only one additional construct, we have not obtained final clones after two attempts.

## Conclusion

We have developed a highly simplified and robust PCR cloning technique termed FastCloning. The new technique eliminates the need of PCR purification/gel purification kit and cloning kit. It is ligation-independent and does not require specific sequence in the vector. Thus, one can insert a DNA fragment into a vector at any desired position without considering restriction sites. This feature also makes it extremely easy to make constructs for fusion proteins and chimeras. In addition, it can be used to make short insertions and multiple mutations spanning a wide region (up to 120 bp) in a cDNA. Finally, it is a highly efficient and reproducible method.

## List of abbreviations

PCR: polymerase chain reaction; nAChR: nicotinic acetylcholine receptor; AChBP: acetylcholine binding protein; CHRNA9: gene name of the human nAChR α9 subunit; CHRNA4: gene name of the human nAChR α4 subunit; CHRNB2: gene name of the human nAChR β2 subunit; 5-HT_3_A: serotonin receptor type 3 A subunit; HTR3A: the gene name for human 5-HT_3_A receptor subunit; ECSM2: endothelial cell specific molecule 2 (also named ECSCR: endothelial cell-specific chemotaxis regulator); AChBP: acetylcholine binding protein; Akt3v1/v2: protein kinase B gamma variant 1 or 2; ZACN: gene name of the human zinc activated cation channel

## Authors' contributions

YC designed the experiment and most primers, and wrote the manuscript. CL performed cloning of human α9 and β2 nAChR subunits into pGEMHE vector, and optimized experimental conditions. He also cloned Akt3v1 or Akt3v2 into pLXSN vector and made a multiple mutation (8 amino acid residue substitutions spanning 13 codons) and several chimeras. AW performed cloning of human 5-HT3A receptor subunit and *APBB1 *into pGEMHE vector and made a quintuple mutant (5 consecutive mutations) in human 5-HT3A subunit. BS performed cloning of human α4 nicotinic receptor subunit and ECSM2 into pGEMHE vector. JL and YH designed primers for ECSM2 and cloned the ECSM2 into p3xFLAG-CMV-14 vector. YH also contributed to manuscript writing and revision. All authors read and approved the final manuscript.
